# compleasm: a faster and more accurate reimplementation of BUSCO

**DOI:** 10.1093/bioinformatics/btad595

**Published:** 2023-09-27

**Authors:** Neng Huang, Heng Li

**Affiliations:** Department of Data Sciences, Dana-Farber Cancer Institute, Boston, MA 02215, United States; Department of Biomedical Informatics, Harvard Medical School, Boston, MA 02115, United States; Department of Data Sciences, Dana-Farber Cancer Institute, Boston, MA 02215, United States; Department of Biomedical Informatics, Harvard Medical School, Boston, MA 02115, United States

## Abstract

**Motivation:**

Evaluating the gene completeness is critical to measuring the quality of a genome assembly. An incomplete assembly can lead to errors in gene predictions, annotation, and other downstream analyses. Benchmarking Universal Single-Copy Orthologs (BUSCO) is a widely used tool for assessing the completeness of genome assembly by testing the presence of a set of single-copy orthologs conserved across a wide range of taxa. However, BUSCO is slow particularly for large genome assemblies. It is cumbersome to apply BUSCO to a large number of assemblies.

**Results:**

Here, we present compleasm, an efficient tool for assessing the completeness of genome assemblies. Compleasm utilizes the miniprot protein-to-genome aligner and the conserved orthologous genes from BUSCO. It is 14 times faster than BUSCO for human assemblies and reports a more accurate completeness of 99.6% than BUSCO’s 95.7%, which is in close agreement with the annotation completeness of 99.5% for T2T-CHM13.

**Availability and implementation:**

https://github.com/huangnengCSU/compleasm.

## 1 Introduction

With the recent advances in sequencing technologies, especially long-read sequencing, genome assembly has undergone a revolution in terms of quality and completeness. Long-read sequencing technologies have improved genome assembly by enabling the generation of more complete and complex genome assemblies, including the telomere-to-telomere assemblies and haplotype-resolved assemblies, which were previously challenging to assemble using short-read sequencing (Wenger *et al.* 2019, Nurk *et al.* 2022). With the development of new assembly algorithms and improvements in computational resources, the time required to assemble a human genome has decreased significantly. It is now possible to assemble a human genome within 8 h (Cheng *et al.* 2021, 2022).

Evaluating assembly completeness is a critical process that provides valuable information about the accuracy and reliability of the assembly and allows for finding potential errors such as missing or misassembled regions. Various tools have been developed for evaluating the completeness of genome assemblies. QUAST (Gurevich *et al.* 2013) is a widely used assembly quality evaluation tool by mapping the contigs/scaffolds of the assemblies to the reference genome and then reports the number of misassemblies, gaps, and various contiguity metrics including N50 and L50, but it is not applicable to new species. Benchmarking Universal Single-Copy Orthologs (BUSCO) is a popular tool for evaluating the completeness of assemblies (Simão *et al.* 2015, Manni *et al.* 2021). BUSCO utilizes a set of conserved single-copy orthologs that are expected to be present in the assemblies and employs gene predictors to confirm the presence of these genes in the assembly to assess the assembly completeness. However, BUSCO may underestimate completeness. For example, for the telomere-to-telomere (T2T) CHM13 assembly (Nurk *et al.* 2022), BUSCO reports a completeness of only 95.7%, but applying BUSCO to annotated protein-coding genes gives a completeness of 99.5%. In addition, BUSCO is inefficient. Assessing the completeness of a human genome assembly using BUSCO can take around 7 h, which is close to the time required for assembling a human genome. With the increasing complexity and size of genome assemblies, there is an urgent need for more efficient and accurate completeness evaluation tools.

In this study, we will introduce compleasm, pronounced/kəmˈplēzəm/, an efficient tool for assessing assembly completeness. It reimplements some logic behind BUSCO but replaces the core protein-to-genome alignment algorithm with miniprot (Li 2023). The evaluation of real datasets demonstrates that compleasm has a significant speed-up compared to BUSCO while achieving higher accuracy.

## 2 Materials and methods

Compleasm first downloads the corresponding BUSCO lineage dataset from https://busco-data.ezlab.org/v5/data (Manni *et al.* 2021, Zdobnov *et al.* 2021) to evaluate the input genome or assembly. Each lineage dataset includes hundreds to over ten thousand near-universally distributed single-copy gene groups with each group consisting of multiple protein sequences from different species (Simão *et al.* 2015). To evaluate the completeness of an input assembly, we map the protein sequences of these single-copy gene groups to the assembly and quantify the occurrences of these single-copy genes in the assembly. In cases the assembly is incomplete, certain single-copy genes cannot be aligned. On the contrary, if the assembly contains falsely duplicated genome regions, some single-copy genes will be aligned to multiple positions in the assembly. Miniprot is a fast aligner for mapping protein sequences to the genome. Compared to similar tools, miniprot has the advantages of shorter runtime and accurate detection of splice junctions and frameshifts. Compleasm performs one round of miniprot, while BUSCO performs two rounds of MetaEuk (Levy Karin *et al.* 2020) with different parameters for high enough sensitivity.

Due to the similarity of sequences in the genome, even if some regions are missing in the assembly, the protein sequences of the corresponding single-copy genes may be aligned to other paralogs in the assembly at lower identity. This may overestimate the completeness. Like BUSCO, we employ HMMER3 (Mistry *et al.* 2013) to confirm orthology. Only matches above the score cutoff defined in lineage files will be retained. Since each single-copy gene may have multiple protein sequences, we choose the protein sequence with the highest hmmersearch score to represent the single-copy gene group.

With paralogous gene matches filtered out, the remaining matches are categorized into one of four types: complete and single-copy, complete and duplicated, fragmented, or missing. A gene is considered missing if it has no alignment after HMMER filtration. It is fragmented if all its alignments are shorter than a length threshold defined by BUSCO. The rest of genes are considered complete. A complete gene is considered to have a single-copy in the assembly if it only has one alignment, or duplicated if it has multiple alignments. Compleasm reports the proportion of genes falling into each of the four categories as the assessment of assembly completeness.

## 3 Results

To evaluate the performance of compleasm, we compared it with BUSCO on various species, including seven reference genomes of model organisms: *Homo sapiens*, *Mus musculus*, *Arabidopsis thaliana*, *Zea mays*, *Drosophila melanogaster*, *Caenorhabditis elegans*, and *Saccharomyces cerevisiae*. Furthermore, we evaluated compleasm and BUSCO on 81 PacBio HiFi assemblies of Metazoa and 22 PacBio HiFi assemblies of Viridiplantae obtained from the Darwin Tree of Life Project. To analyze the frameshifts reported by compleasm, we ran compleasm on several Nanopore assemblies and PacBio assemblies. The details of the datasets can be found in Supplementary Table S1. For BUSCO, we used the option “-m genome” and the default gene predictor MetaEuk.

### 3.1 Evaluation of model organism reference genomes

Table 1 shows the comparison of compleasm and BUSCO on the reference genomes of seven model organisms. For *A.thaliana* and *S.cerevisiae* reference, the results of compleasm and BUSCO shows a difference of completeness (single-copy and duplicated) <1%. For *D.melanogaster*, *C.elegans*, and *M.musculus*, the differences between the assessment results of compleasm and BUSCO range from 1% to 3%. When evaluating the reference genomes of *H.sapiens* and *Z.mays*, compleasm and BUSCO show a large difference exceeding 3%. For most reference genomes, compleasm reports higher completeness than BUSCO.

**Table 1.** Comparison of compleasm and BUSCO on the reference genomes of seven model organisms.

Model organism	Lineage	Tools	Completed (%)	Single-copy (%)	Duplicated (%)	Fragmented (%)	Missing (%)	*N*
*A.thaliana*	brassicales_odb10	compleasm	99.9	98.9	1.0	0.1	0.0	4596
		BUSCO	99.2	97.9	1.3	0.1	0.7	4596
*D.melanogaster*	diptera_odb10	compleasm	99.7	99.4	0.3	0.2	0.1	3285
		BUSCO	98.6	98.4	0.2	0.5	0.9	3285
*S.cerevisiae*	saccharomycetes_odb10	compleasm	99.2	97.6	1.6	0.3	0.5	2137
		BUSCO	99.5	97.3	2.2	0.1	0.4	2137
*H.sapiens*	primates_odb10	compleasm	99.6	98.9	0.7	0.3	0.1	13 780
		BUSCO	95.7	94.1	1.6	1.1	3.2	13 780
*C.elegans*	nematoda_odb10	compleasm	99.8	99.7	0.1	0.2	0.0	3131
		BUSCO	98.8	98.3	0.5	0.6	0.6	3131
*M.musculus*	glires_odb10	compleasm	99.7	97.8	1.9	0.3	0.0	13 798
		BUSCO	96.5	93.6	2.9	0.6	2.9	13 798
*Z.mays*	liliopsida_odb10	compleasm	96.7	82.2	14.5	3.0	0.3	3236
		BUSCO	93.8	79.2	14.6	5.3	0.9	3236

In the assessment of the *H.sapiens* reference genome (T2T-CHM13), compleasm reported completeness of 99.6% compared to 95.7% reported by BUSCO. Among them, 562 complete genes were only reported by compleasm. We subsequently evaluated these compleasm-specific genes using the annotation of T2T-CHM13 from NCBI. By comparing the alignments of the complete genes with the annotation, we deemed a complete gene to be supported by annotation if the sum of overlap length in codons between the complete gene and annotation exceeds 50% of the total length of codons in annotation (see Supplementary Fig. S1). We found that 557 out of 562 compleasm-specific complete genes could be supported by the annotation.

Additionally, we used the “protein” mode of BUSCO to assess the completeness of annotated T2T-CHM13 genes with the same lineage “primate_odb10.” We obtained the protein sequences of the T2T-CHM13 from NCBI and assessed the completeness with the command “*busco -m protein -i INPUT.amino_acids -o OUTPUT -l LINEAGE*.” In the protein mode, BUSCO searches the proteins of annotated gene set in the sequence database of the lineage dataset using HMMER3 (Mistry *et al.* 2013). Supplementary Table S2 shows the assessment result of the annotated gene set, Complete: 99.5% (Single: 28.9%, Duplicated: 70.6%), Fragmented: 0%, Missing: 0.5%. The completeness of annotated gene set is similar to the completeness of 99.6% obtained by compleasm but differs from the completeness of 95.7% from BUSCO. By tracking the procedure of hmmsearch of these false negative complete genes in BUSCO, we observed that the protein sequences derived from the predicted genes by MetaEuk failed to meet the score thresholds. This indicates that these translated protein sequences have low quality, likely due to the errors in the protein-to-genome alignment. Supplementary Figure S2 shows the protein-to-genome alignment of one of the compleasm-specific complete genes. From that figure, we can see that some protein sequence fragments are missing in the MetaEuk alignment, while miniprot aligns the protein sequence almost completely to the CHM13 genome, and the alignment is supported by the CHM13 annotation. The missing fragments by MetaEuk may be because MetaEuk cannot find the exact splice junction. Furthermore, due to the presence of alternative splicing in the protein sequences of T2T-CHM13 annotated genes, numerous genes are reported as complete and duplicated by BUSCO. For the *Z.mays* reference genome, compleasm and BUSCO reported proportions of complete genes at 96.7% and 93.8%, respectively. We also evaluated the completeness of the annotation gene set of *Z.mays* (Supplementary Table S3) and the completeness of the annotation gene set is 96.8%, which is similar to the value obtained by compleasm.

### 3.2 Evaluating the specificity of compleasm

To check if compleasm is overcalling complete genes, we removed chromosome 1 from T2T-CHM13 reference and compared the completeness of the modified CHM13 genome without chromosome 1 to the completeness of the entire CHM13 genome. Supplementary Table S4 presents the assessment results by compleasm and BUSCO. The completeness of T2T-CHM13 genome without chromosome 1 reported by compleasm is 89.9% while the value reported by BUSCO is 86.2%. Deletion of chromosome 1 reduced the completeness of T2T-CHM13 by 9.7% and 9.5% in compleasm and BUSCO assessments, respectively. On the entire CHM13, compleasm reported 13 622 complete single-copy genes, 1435 of them were mapped to chromosome 1. After we removed chromosome 1 and ran compleasm on the remaining chromosomes, compleasm still reported 93 of them as complete single-copy genes. Checking the alignment of these 93 genes, we found that 60 of them were processed pseudogenes. As for the remaining 33 genes, their hits on other chromosomes were not as good as hits to chromosome 1 and suppressed by miniprot as miniprot was tuned to ignore alignments whose scores were below 97% of the best alignment scores. With chromosome 1 removed, the hits of genes to other chromosomes surfaced, passed the HMMER thresholds and became single-copy genes. Overall, the great majority of complete single-copy genes reported by compleasm were real.

### 3.3 Evaluation of runtime

**Figure 1.**
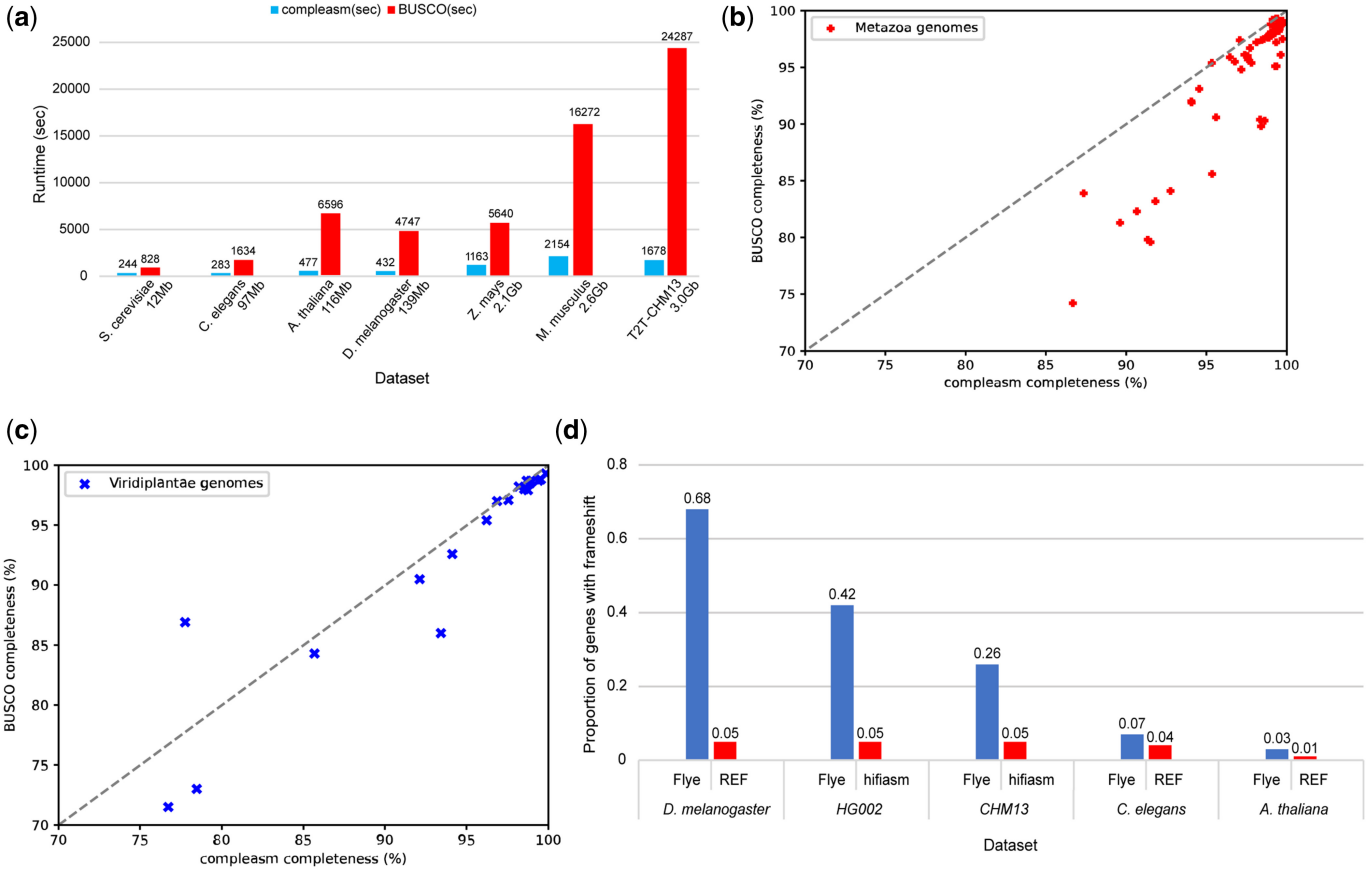
(a) Comparison of the runtime of compleasm and BUSCO on real datasets. (b) The comparison of completeness reported by compleasm and BUSCO on HiFi assemblies of Metazoa genomes. (c) The comparison of completeness reported by compleasm and BUSCO on HiFi assemblies of Viridiplantae genomes. (d) The proportions of BUSCO genes with frameshifts reported by compleasm on datasets of different species.

Figure 1a shows the runtimes of compleasm and BUSCO on the seven reference genomes. All experiments were conducted on a single server with 40 threads. Notably, compleasm achieves significant improvement over BUSCO by 3.4–14.5 times. Moreover, the speedup tends to increase with larger genome sizes, highlighting the efficiency of compleasm in handling larger genomes. The evaluation of *H.sapiens* genome reduces from 6.7 to 0.4 h. During the evaluation process of both compleasm and BUSCO, the main time consumption is protein-to-genome alignment (MetaEuk/miniprot) and hmmsearch. There are two rounds of MetaEuk+hmmsearch in BUSCO while compleasm only has one round of miniprot+hmmsearch. Meanwhile, miniprot has a 10-fold speedup over MetaEuk (Li 2023). In summary, compleasm can greatly improve the speed of evaluation. Furthermore, when dealing with small genomes, the evaluation process’s runtime is primarily influenced by hmmsearch, which relies on the size of the lineage dataset. However, for larger genomes, the running time of the evaluation process is mainly determined by protein-to-genome alignment. As the genome size increases, compleasm demonstrates superior acceleration compared to BUSCO.

### 3.4 Evaluation of 103 HiFi assemblies of Metazoa and Viridiplantae

In addition to the reference genomes, we evaluated the completeness of 103 PacBio HiFi assemblies using compleasm. The BUSCO assessments of these assemblies were downloaded from the website of Blobtoolkit2 (https://blobtoolkit.genomehubs.org) (Challis *et al.* 2020). Supplementary Tables S5 and S6 present the assessment results of 81 Metazoa assemblies and 22 Viridiplantae assemblies, respectively. Figure 1b shows the numbers of completed genes reported by compleasm and BUSCO on HiFi assemblies of Metazoa genomes. On 78 out of 81 Metazoa assemblies, compleasm reports a higher completeness range of 0%–12% than BUSCO. For the remaining three Metazoa assemblies, there is a slight difference of <0.3% in completeness between compleasm and BUSCO. Figure 1c presents the comparison of the completeness reported by compleasm and BUSCO on HiFi assemblies of Viridiplantae genomes. On 20 out of 22 Viridiplantae assemblies, compleasm obtained a higher completeness range of 0%–7.5% than BUSCO. However, on the genome of *Pycnococcus provasolii*, compleasm reported completeness that was 7.5% lower than that reported by BUSCO. By tracking the protein alignment of this genome, we found that most of the alignment records have very low identities due to the high divergence. Therefore, we add a feature to compleasm to ensure a more reliable assessment of assembly completeness for input assemblies with high divergence. If the average identity of protein alignments for all BUSCO genes is lower than 50%, compleasm will print a message and recommend running BUSCO additionally.

### 3.5 Frameshift analysis

Frameshift refers to the insertion or deletion of several base pairs that are not a multiple of three, disrupting the open reading frame. Miniprot can align through frameshifts in the genome sequence and identify them. Compleasm outputs the number of frameshifts in each mapped gene by parsing the cigar sequences in the alignment output file. The number of frameshifts can be used as a measure of the quality of an assembly. We ran compleasm and calculated the percentage of BUSCO genes with frameshifts on the assemblies or reference genomes of different species. We conducted a comparison of frameshifts in Flye assemblies and hifiasm assemblies for the genomes of HG002 and CHM13. The Flye assemblies of HG002 and CHM13 were assembled from 110× to 120× Nanopore reads by Flye (Kolmogorov *et al.* 2019) and the hifiasm assemblies of HG002 and CHM13 were generated from 32× to 36× PacBio HiFi reads by hifiasm (Cheng *et al.* 2021, 2022). Figure 1d shows the proportions of BUSCO genes with frameshifts in Flye assemblies of HG002 and CHM13 are 0.42 and 0.26, respectively, whereas both hifiasm assemblies demonstrate a lower value of 0.05. The Nanopore assemblies had a much higher frameshift error rate. We had a similar observation for *D.melanogaster*, *C.elegans*, and *A.thaliana*, assembled from 30× Nanopore reads, 40× PacBio CLR reads and 75× PacBio CLR reads, respectively. Nonetheless, the completeness assessments reported by compleasm were not affected by the high error rate much (Supplementary Table S7) because miniprot can align through frameshifts. On the contrary, the completeness reported by BUSCO decreased because MetaEuk interrupted the sequence alignment due to frameshifts. Separating frameshift errors from assembly incompleteness is a unique advantage of compleasm.

## 4 Discussion

This study shows that compleasm is a highly efficient tool for evaluating the completeness of genome assemblies, offering improved accuracy and reduced evaluation time. However, it should be noted that compleasm has limited sensitivity to distant homologs. Therefore, for assemblies with high divergence, combining the results from compleasm and BUSCO is recommended to ensure higher reliability.

## Supplementary Material

btad595_Supplementary_Data

## References

[btad595-B1] Challis R , Richards E, Rajan J et al Blobtoolkit—interactive quality assessment of genome assemblies. G3 (Bethesda) 2020;10:1361–74. 32071071 10.1534/g3.119.400908 PMC7144090

[btad595-B2] Cheng H , Concepcion GT, Feng X et al Haplotype-resolved de novo assembly using phased assembly graphs with hifiasm. Nat Methods 2021;18:170–5. 33526886 10.1038/s41592-020-01056-5 PMC7961889

[btad595-B3] Cheng H , Jarvis ED, Fedrigo O et al Haplotype-resolved assembly of diploid genomes without parental data. Nat Biotechnol 2022;40:1332–5. 35332338 10.1038/s41587-022-01261-x PMC9464699

[btad595-B4] Gurevich A , Saveliev V, Vyahhi N et al QUAST: quality assessment tool for genome assemblies. Bioinformatics 2013;29:1072–5. 23422339 10.1093/bioinformatics/btt086 PMC3624806

[btad595-B5] Kolmogorov M , Yuan J, Lin Y et al Assembly of long, error-prone reads using repeat graphs. Nat Biotechnol 2019;37:540–6. 30936562 10.1038/s41587-019-0072-8

[btad595-B6] Levy Karin E , Mirdita M, Söding J. Metaeuk-sensitive, high-throughput gene discovery, and annotation for large-scale eukaryotic metagenomics. Microbiome, 2020;8:48. 32245390 10.1186/s40168-020-00808-x PMC7126354

[btad595-B7] Li H. Protein-to-genome alignment with miniprot. Bioinformatics 2023;39:btad014. 36648328 10.1093/bioinformatics/btad014 PMC9869432

[btad595-B8] Manni M , Berkeley MR, Seppey M et al Busco update: novel and streamlined workflows along with broader and deeper phylogenetic coverage for scoring of eukaryotic, prokaryotic, and viral genomes. Mol Biol Evol 2021;38:4647–54. 34320186 10.1093/molbev/msab199 PMC8476166

[btad595-B9] Mistry J , Finn RD, Eddy SR et al Challenges in homology search: HMMER3 and convergent evolution of coiled–coil regions. Nucleic Acids Res 2013;41:e121. 23598997 10.1093/nar/gkt263 PMC3695513

[btad595-B10] Nurk S , Koren S, Rhie A et al The complete sequence of a human genome. Science 2022;376:44–53. 35357919 10.1126/science.abj6987 PMC9186530

[btad595-B11] Simão FA , Waterhouse RM, Ioannidis P et al Busco: assessing genome assembly and annotation completeness with single-copy orthologs. Bioinformatics 2015;31:3210–2. 26059717 10.1093/bioinformatics/btv351

[btad595-B12] Wenger AM , Peluso P, Rowell WJ et al Accurate circular consensus long-read sequencing improves variant detection and assembly of a human genome. Nat Biotechnol 2019;37:1155–62. 31406327 10.1038/s41587-019-0217-9 PMC6776680

[btad595-B13] Zdobnov EM , Kuznetsov D, Tegenfeldt F et al OrthoDB in 2020: evolutionary and functional annotations of orthologs. Nucleic Acids Res 2021;49:D389–93. 33196836 10.1093/nar/gkaa1009 PMC7779051

