# Supplementary material for: compleasm: a faster and more accurate reimplementation of BUSCO
Source: Bioinformatics. 2023 Sep 27;39(10):btad595. doi: 10.1093/bioinformatics/btad595 (PMC10558035; doi:10.1093/bioinformatics/btad595)
Supplement: btad595_Supplementary_Data [file btad595_supplementary_data.pdf]

Supplementary Materials

Supplementary Tables

Table S1. The details of datasets.

Dataset	Type	Accession
Arabidopsis thaliana	Reference	GCF_000001735.4_TAIR10.1_genomic.fna.gz
Drosophila melanogaster	Reference	GCF_000001215.4_Release_6_plus_ISO1_MT_genomic.fna.gz
Saccharomyces cerevisiae	Reference	GCF_000146045.2_R64_genomic.fna.gz
Homo sapiens	Reference	GCF_009914755.1_T2T-CHM13v2.0_genomic.fna.gz
Caenorhabditis elegans	Reference	GCF_000002985.6_WBcel235_genomic.fna.gz
Mus musculus	Reference	GCF_000001635.27_GRCm39/GCF_000001635.27_GRCm39_genomic.fna.gz
Zea mays	Reference	GCF_902167145.1_Zm-B73-REFERENCE-NAM-5.0_genomic.fna.gz
Metazoa	HiFi assemblies	https://portal.darwintreeoflife.org/data
Viridiplantae	HiFi assemblies	https://portal.darwintreeoflife.org/data
CHM13	HiFi assembly	CHM13.HiFi.hifiasm-0.12.fa.gz
CHM13	ONT assembly	flye.v29.chm13.ont.120x.fasta.gz
HG002	HiFi assembly	NA24385.HiFi.hifiasm-0.12.pri.fa.gz
HG002	ONT assembly	flye.v29.hg002.ont.110x.fasta.gz
Drosophila melanogaster	ONT assembly	flye.v29.dmelanogaster.ont.30x.fasta.gz
Caenorhabditis elegans	PacBio CLR assembly	flye.v29.celegans.pb.40x.fasta.gz
Arabidopsis thaliana	PacBio CLR assembly	flye.v29.athaliana.pb.75x.fasta.gz

Table S2. The completeness of annotated gene set of T2T-CHM13 evaluated by BUSCO.

C:99.5% [S:28.9%, D:70.6%], F:0.0%, M:0.5%, n:13780	
Complete (C)	13708
Complete and single-copy (S)	3976
Complete and duplicated (D)	9732
Fragmented (F)	4
Missing (M)	68
Total (n)	13780

Table S3. The completeness of annotated gene set of reference genome *Z. mays* evaluated by BUSCO.

C:96.8% [S:53.1%, D:43.7%], F:1.6%, M:1.6%, n:3236	
Complete (C)	3133
Complete and single-copy (S)	1718
Complete and duplicated (D)	1415
Fragmented (F)	53
Missing (M)	50
Total (n)	3236

Table S4. The assessment of completeness of the entire T2T-CHM13 genome and the modified T2T-CHM13 without chromosome 1.

Dataset	Tool	Completed (%)	Single-copy (%)	Duplicated (%)	Fragmented (%)	Missing (%)	N
T2T-CHM13	compleasm	99.6	98.9	0.7	0.3	0.1	13780
	BUSCO	95.7	94.1	1.6	1.1	3.2	13780
T2T-CHM13-without-chr1	compleasm	89.9	89.2	0.7	0.4	9.7	13780
	BUSCO	86.2	84.7	1.5	1.0	12.8	13780

Table S5. The assessment of completeness of 81 HiFi assemblies of Metazoa by compleasm and BUSCO

Accession	Taxon	Lineage	compleasm					BUSCO				
			Complete (%)	Single (%)	Duplicated (%)	Fragmented (%)	Missing (%)	Complete (%)	Single (%)	Duplicated (%)	Fragmented (%)	Missing (%)
GCA_943735955.1	Piscicola geometra	metazoa	86.7	86.2	0.5	1.8	11.5	74.2	73.5	0.7	9.0	16.8
GCA_922989275.1	Gari tellinella	mollusca	91.5	90.6	0.9	1.0	7.4	79.6	78.4	1.2	4.8	15.7
GCA_947247005.1	Spisula solida	mollusca	91.3	90.5	0.8	1.3	7.5	79.8	78.7	1.1	4.6	15.6
GCA_921293015.1	Phorcus lineatus	mollusca	95.4	94.9	0.5	1.2	3.5	85.6	85.0	0.6	4.4	10.0
GCA_935421135.1	Bugulina stolonifera	metazoa	92.8	92.5	0.3	1.3	6.0	84.1	83.1	0.9	7.0	8.9
GCA_945261195.1	Cryptosula pallasiana	metazoa	91.8	91.5	0.3	1.8	6.4	83.2	82.1	1.2	8.0	8.8
GCA_929108145.1	Limnephilus rhombicus	endopterygota	98.4	97.6	0.8	0.8	0.9	89.8	88.6	1.2	7.1	3.2
GCA_936435175.1	Glyphotaelius pellucidus	endopterygota	98.6	98.1	0.6	0.7	0.7	90.3	89.5	0.8	6.8	2.9
GCA_916610825.1	Haliclystus octoradiatus	metazoa	89.6	89.4	0.2	1.7	8.7	81.3	81.1	0.2	9.4	9.2
GCA_914767715.1	Membranipora membranacea	metazoa	90.6	89.3	1.3	1.9	7.6	82.3	79.9	2.4	8.3	9.4
GCA_917880885.1	Limnephilus marmoratus	endopterygota	98.4	97.7	0.7	0.8	0.9	90.4	89.3	1.1	6.7	2.9
GCA_945859605.1	Lumbricus rubellus	metazoa	95.6	93.7	1.9	0.9	3.5	90.6	86.9	3.7	4.6	4.8
GCA_922984935.1	Meles meles	carnivora	99.4	97.6	1.8	0.4	0.3	95.1	93.2	2.0	0.8	4.0
GCA_922990625.1	Meles meles	carnivora	99.3	97.7	1.6	0.4	0.3	95.1	93.0	2.1	0.8	4.1
GCA_910594005.1	Cervus elaphus	cetartiodactyla	99.6	97.1	2.5	0.3	0.1	96.1	92.8	3.3	1.1	2.8
GCA_916048095.1	Sacculina carcini	arthropoda	87.4	87.0	0.4	1.4	11.3	83.9	83.2	0.7	6.1	10.0
GCA_942159475.1	Sthenelais limicola	metazoa	97.8	97.0	0.8	0.6	1.6	95.4	94.3	1.0	2.8	1.8
GCA_932526225.1	Nematostella vectensis	metazoa	97.2	96.9	0.3	0.4	2.4	94.8	94.2	0.5	2.5	2.7
GCA_929443795.1	Accipiter gentilis	aves	99.7	99.4	0.3	0.2	0.1	97.5	96.7	0.8	0.6	1.9
GCA_946894095.1	Incurvaria masculella	lepidoptera	94.1	93.7	0.4	0.7	5.3	91.9	91.1	0.8	1.4	6.7
GCA_921293095.1	Ischnura elegans	insecta	99.3	98.7	0.6	0.3	0.4	97.2	96.4	0.8	1.3	1.5
GCA_946902875.1	Nematopogon swammerdamellus	lepidoptera	94.1	93.5	0.5	0.7	5.3	92.0	90.9	1.1	1.3	6.7
GCA_936440205.1	Lepidonotus clava	metazoa	97.6	97.4	0.2	0.6	1.8	95.7	95.3	0.4	2.3	2.0
GCA_918808275.1	Dolichovespula sylvestris	hymenoptera	97.6	95.9	1.7	0.9	1.6	96.0	95.7	0.3	1.0	3.0
GCA_918807975.1	Aplidium turbinatum	metazoa	94.6	92.1	2.4	1.3	4.2	93.1	88.8	4.3	3.4	3.6
GCA_947063395.1	Hemistola chrysoprasaria	lepidoptera	99.5	99.2	0.3	0.3	0.2	98.2	97.8	0.4	0.4	1.3
GCA_947310845.1	Yponomeuta plumbellus	lepidoptera	98.9	98.7	0.2	0.3	0.8	97.6	97.2	0.4	0.7	1.7
GCA_918843875.1	Diadumene lineata	metazoa	97.4	97.2	0.2	0.7	1.9	96.1	95.6	0.5	2.0	1.9
GCA_947458855.1	Monopis laevigella	lepidoptera	96.8	96.2	0.6	0.6	2.7	95.5	94.6	0.9	0.8	3.7
GCA_923062675.1	Marasmarcha lunaedactyla	lepidoptera	98.8	98.2	0.6	0.3	0.8	97.6	96.7	0.9	0.7	1.7
GCA_934047225.1	Ypsolopha sequella	lepidoptera	99.2	98.5	0.7	0.3	0.5	98.0	96.9	1.2	0.5	1.5
GCA_947086465.1	Eupithecia exiguata	lepidoptera	99.1	98.9	0.3	0.3	0.6	97.9	97.4	0.5	0.6	1.5
GCA_947172395.1	Bicyclus anynana	lepidoptera	99.4	98.9	0.5	0.3	0.3	98.2	97.6	0.6	0.4	1.4
GCA_947049275.1	Epinotia bilunana	lepidoptera	99.1	98.8	0.3	0.3	0.6	97.9	97.4	0.5	0.6	1.6
GCA_934044485.1	Apeira syringaria	lepidoptera	99.6	99.0	0.6	0.3	0.2	98.4	97.7	0.7	0.5	1.0
GCA_947507515.1	Eulithis testata	lepidoptera	99.3	99.2	0.1	0.3	0.4	98.1	97.8	0.4	0.5	1.4
GCA_947044415.1	Eupithecia dodoneata	lepidoptera	99.1	98.9	0.3	0.3	0.5	98.0	97.6	0.4	0.5	1.4
GCA_932527185.1	Ecliptopera silaceata	lepidoptera	99.4	99.1	0.3	0.3	0.3	98.3	97.9	0.4	0.4	1.3
GCA_910589615.1	Taurulus bubalis	actinopterygii	99.5	99.1	0.4	0.4	0.2	98.4	97.6	0.8	0.5	1.1
GCA_934045075.1	Yponomeuta sedellus	lepidoptera	98.6	98.2	0.4	0.3	1.1	97.5	97.0	0.5	0.6	1.9
GCA_933210815.1	Meta bourneti	arachnida	99.0	95.6	3.3	0.2	0.8	97.9	92.2	5.7	0.8	1.3
GCA_947359385.1	Chesias legatella	lepidoptera	99.5	99.2	0.3	0.2	0.3	98.4	98.0	0.4	0.5	1.2
GCA_921293045.1	Aplocera efformata	lepidoptera	99.5	99.3	0.2	0.3	0.3	98.4	98.1	0.3	0.4	1.2
GCA_947310995.1	Yponomeuta cagnagella	lepidoptera	98.9	98.6	0.3	0.4	0.8	97.8	97.4	0.4	0.5	1.8
GCA_932526625.1	Rhingia campestris	diptera	98.4	97.5	0.9	0.5	1.1	97.4	95.9	1.4	0.8	1.8
GCA_946894065.1	Acleris literana	lepidoptera	99.1	98.8	0.4	0.3	0.6	98.1	97.4	0.7	0.4	1.5
GCA_945859705.1	Episyrphus balteatus	diptera	97.7	97.3	0.5	0.7	1.6	96.7	96.0	0.7	0.7	2.6
GCA_923062465.1	Acleris sparsana	lepidoptera	99.3	99.0	0.4	0.2	0.5	98.3	97.7	0.6	0.4	1.3
GCA_947284805.1	Coleophora flavipennella	lepidoptera	98.9	98.2	0.8	0.3	0.8	97.9	96.8	1.1	0.5	1.7
GCA_947369235.1	Udea olivalis	lepidoptera	99.6	99.3	0.4	0.3	0.1	98.7	98.4	0.4	0.3	1.0
GCA_905147045.1	Nymphalis io	lepidoptera	99.7	99.3	0.4	0.3	0.0	98.8	98.5	0.3	0.4	0.9
GCA_947086405.1	Esperia sulphurella	lepidoptera	99.0	98.5	0.5	0.3	0.7	98.1	97.5	0.7	0.3	1.6
GCA_946965025.1	Blera fallax	diptera	98.1	97.8	0.4	0.6	1.3	97.2	96.8	0.4	0.8	2.0
GCA_922987775.1	Agonopterix subpropinquella	lepidoptera	99.6	99.0	0.7	0.2	0.2	98.7	97.9	0.7	0.2	1.2
GCA_947044815.1	Apomyelois bistriatella	lepidoptera	99.7	99.4	0.3	0.3	0.0	98.8	98.3	0.5	0.3	0.9
GCA_947034925.1	Eudemis profundana	lepidoptera	99.4	98.7	0.7	0.3	0.4	98.5	97.4	1.1	0.4	1.1
GCA_921293315.1	Nemurella pictetii	insecta	99.6	99.0	0.7	0.2	0.2	98.8	98.0	0.8	0.4	0.8
GCA_947508005.1	Amphipoea lucens	lepidoptera	99.5	99.2	0.3	0.2	0.3	98.7	98.1	0.6	0.3	1.0
GCA_945859575.1	Herminia tarsipennalis	lepidoptera	99.4	98.7	0.7	0.2	0.3	98.6	97.7	0.9	0.3	1.1
GCA_905146925.1	Autographa gamma	lepidoptera	99.6	99.4	0.3	0.3	0.1	98.8	98.6	0.3	0.2	0.9
GCA_932526445.1	Phragmatobia fuliginosa	lepidoptera	99.5	98.9	0.6	0.3	0.2	98.7	97.9	0.8	0.3	1.0
GCA_936440315.1	Barbus barbus	actinopterygii	99.5	20.5	79.0	0.4	0.1	98.7	21.0	77.6	0.5	0.8
GCA_927399405.1	Agonopterix arenella	lepidoptera	99.5	99.0	0.5	0.2	0.3	98.7	98.0	0.7	0.3	1.0
GCA_947462355.1	Caradrina kadenii	lepidoptera	99.6	99.5	0.1	0.3	0.1	98.9	98.6	0.3	0.3	0.8
GCA_921972225.1	Euplexia lucipara	lepidoptera	99.7	99.5	0.3	0.2	0.0	99.0	98.5	0.5	0.3	0.7
GCA_918843915.1	Notodonta ziczac	lepidoptera	99.6	99.2	0.4	0.3	0.2	98.9	98.4	0.4	0.3	0.9
GCA_947347705.1	Nymphula nitidulata	lepidoptera	99.6	99.4	0.2	0.3	0.2	98.9	98.6	0.3	0.2	0.9
GCA_934045905.1	Leuctra nigra	insecta	99.6	98.0	1.6	0.2	0.3	98.9	97.0	1.9	0.5	0.6
GCA_947256265.1	Acronicta leporina	lepidoptera	99.8	99.6	0.1	0.2	0.0	99.1	98.8	0.3	0.2	0.7
GCA_947361185.1	Charanyca ferruginea	lepidoptera	99.6	96.7	2.8	0.2	0.2	98.9	95.9	3.0	0.2	0.9
GCA_947363495.1	Euzophera pinguis	lepidoptera	99.5	99.2	0.3	0.3	0.2	98.9	98.5	0.3	0.3	0.8
GCA_916618015.1	Xestia c-nigrum	lepidoptera	99.4	99.1	0.4	0.3	0.3	98.8	98.2	0.5	0.2	1.0
GCA_921293005.1	Nemoura dubitans	insecta	99.4	98.5	0.9	0.2	0.4	98.8	97.7	1.2	0.4	0.7
GCA_905475395.1	Chrysoperla carnea	endopterygota	96.4	96.0	0.5	0.8	2.8	95.9	95.0	0.9	1.0	3.1
GCA_944039245.1	Nebria salina	endopterygota	99.0	98.9	0.1	0.7	0.3	98.8	98.4	0.3	0.7	0.5
GCA_933228885.1	Leistus spinibarbis	endopterygota	99.3	99.2	0.1	0.5	0.1	99.2	98.7	0.4	0.5	0.3
GCA_933228675.1	Stomorhina lunata	diptera	99.4	99.0	0.5	0.4	0.2	99.3	98.8	0.5	0.2	0.5
GCA_911728435.1	Apoderus coryli	endopterygota	99.3	98.4	1.0	0.4	0.2	99.3	97.7	1.6	0.4	0.3
GCA_922984085.1	Sicus ferrugineus	diptera	95.3	94.8	0.6	1.0	3.7	95.4	94.3	1.0	0.7	3.9
GCA_930367205.1	Acanthosoma haemorrhoidale	hemiptera	99.1	98.1	1.0	0.1	0.8	99.2	97.4	1.8	0.1	0.7
GCA_914767665.1	Harmonia axyridis	endopterygota	97.1	95.7	1.4	0.8	2.2	97.4	95.2	2.3	0.6	2.0

Table S6. The assessment of completeness of 22 HiFi assemblies of Viridiplantae by compleasm and BUSCO

Accession	Taxon	Lineage	compleasm					BUSCO				
			Complete (%)	Single (%)	Duplicated (%)	Fragmented (%)	Missing (%)	Complete (%)	Single (%)	Duplicated (%)	Fragmented (%)	Missing (%)
GCA_914767535.1	Dunaliella primolecta	chlorophyta	93.4	92.9	0.5	0.3	6.3	86.0	85.4	0.6	4.0	10.0
GCA_946800655.1	Juncus effusus	poales	78.5	75.4	3.1	1.8	19.8	73.0	68.0	5.0	3.0	24.0
GCA_946800325.1	Luzula sylvatica	poales	76.7	72.5	4.2	1.7	21.6	71.5	66.4	5.1	2.8	25.7
GCA_933775445.1	Potentilla anserina	eudicots	92.1	91.1	1.1	1.5	6.4	90.5	88.8	1.7	1.7	7.9
GCA_934048045.1	Polygonum aviculare	eudicots	94.1	90.2	4.0	0.8	5.1	92.6	86.9	5.7	1.8	5.6
GCA_947034895.1	Rhytidiadelphus loreus	embryophyta	85.6	81.0	4.7	3.4	11.0	84.3	77.6	6.7	2.9	12.8
GCA_947034885.1	Thuidium tamariscinum	embryophyta	61.8	58.7	3.1	3.4	34.8	60.5	56.3	4.2	2.7	36.7
GCA_937616625.1	Mercurialis annua	eudicots	98.8	97.4	1.4	0.3	0.9	97.9	95.9	2.0	0.6	1.6
GCA_946814005.1	Chamaenerion angustifolium	eudicots	96.2	82.8	13.5	0.8	3.0	95.4	79.6	15.8	1.1	3.5
GCA_946800305.1	Medicago arabica	fabales	99.6	98.0	1.6	0.2	0.3	98.8	96.6	2.2	0.2	1.0
GCA_946807835.1	Ailanthus altissimus	eudicots	99.4	61.3	38.1	0.3	0.3	98.7	54.7	44.0	0.3	0.9
GCA_933208065.1	Arabidopsis thaliana	brassicales	99.9	98.8	1.0	0.1	0.0	99.3	97.9	1.4	0.1	0.7
GCA_946800315.1	Misopates orontium	eudicots	98.5	96.1	2.5	0.4	1.0	98.0	94.8	3.2	0.3	1.6
GCA_948329865.1	Linaria vulgaris	eudicots	97.6	94.4	3.2	0.7	1.7	97.1	92.6	4.5	0.6	2.3
GCA_916050505.1	Malus domestica	eudicots	98.9	63.2	35.6	0.4	0.7	98.5	59.5	39.0	0.5	1.1
GCA_916048215.1	Malus sylvestris	eudicots	98.9	62.8	36.1	0.4	0.7	98.5	59.4	39.1	0.6	0.9
GCA_916615385.1	Malus domestica	eudicots	99.0	62.9	36.1	0.4	0.6	98.6	59.5	39.1	0.6	0.8
GCA_916612005.1	Malus domestica	eudicots	98.9	62.5	36.4	0.4	0.7	98.7	59.9	38.8	0.5	0.8
GCA_946896805.1	Scutellaria galericulata	eudicots	98.2	94.8	3.4	0.5	1.3	98.2	93.9	4.3	0.2	1.6
GCA_946800695.1	Geum urbanum	eudicots	98.7	25.4	73.3	0.4	0.9	98.7	18.6	80.1	0.3	1.1
GCA_947034835.1	Ballota nigra	eudicots	96.9	94.2	2.7	0.8	2.4	97.0	92.9	4.0	0.3	2.8
GCA_938743325.1	Pycnococcus provasolii	chlorophyta	77.8	76.4	1.4	0.7	21.6	86.9	83.7	3.2	1.1	12.0

Table S7. The assessment of completeness of several datasets of Flye assemblies, hifiasm assemblies and reference genomes by compleasm

Dataset	Lineage	compleasm					BUSCO				
		Completed (%)	Single-copy (%)	Duplicated (%)	Fragmented (%)	Missing (%)	Completed (%)	Single-copy (%)	Duplicated (%)	Fragmented (%)	Missing (%)
HG002-Flye	primate_odb10	99.4	98.7	0.7	0.4	0.2	88.5	86.8	1.7	3.9	7.6
HG002-hifiasm	primate_odb10	99.5	98.7	0.8	0.4	0.2	95.6	93.9	1.7	1.1	3.3
CHM13-Flye	primate_odb10	99.5	98.8	0.7	0.4	0.2	92.0	90.4	1.6	2.9	5.1
CHM13-hifiasm	primate_odb10	99.5	98.8	0.7	0.4	0.1	95.7	94.0	1.7	1.1	3.2
A. thaliana-Flye	brassicales_odb10	99.8	98.8	1.1	0.1	0.0	98.9	97.4	1.5	0.2	0.9
A. thaliana-reference	brassicales_odb10	99.9	98.9	1.0	0.1	0.0	99.2	97.9	1.0	0.1	0.0
C. elegans-Flye	nematoda_odb10	99.8	99.6	0.2	0.2	0.0	98.7	98.2	0.5	0.7	0.6
C. elegans-reference	nematoda_odb10	99.8	99.7	0.1	0.2	0.0	98.8	98.3	0.5	0.6	0.6
D. melanogaster-Flye	diptera_odb10	99.6	99.5	0.1	0.3	0.1	84.8	84.5	0.3	8.6	6.6
D. melanogaster-reference	diptera_odb10	99.7	99.4	0.3	0.2	0.1	98.6	98.4	0.2	0.5	0.9

Supplementary Figures

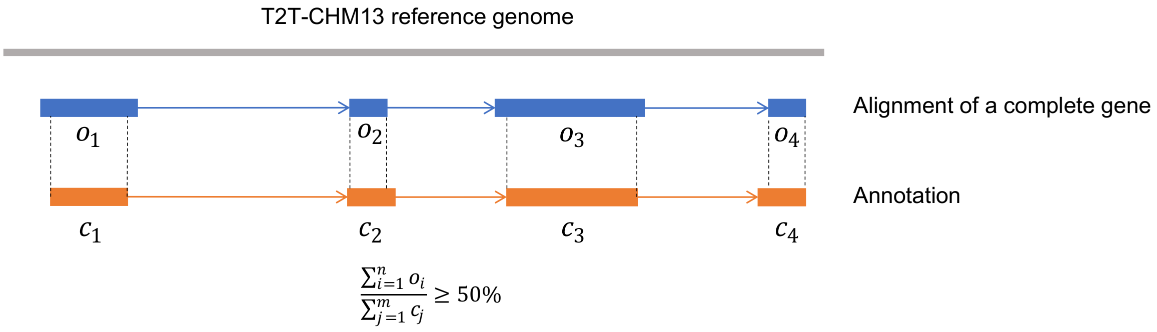

Figure S1. Example of the complete gene confirmed by the annotation. o_i is the length of overlaps in codons between complete gene and annotation. c_j is the length of a codon in the annotation.

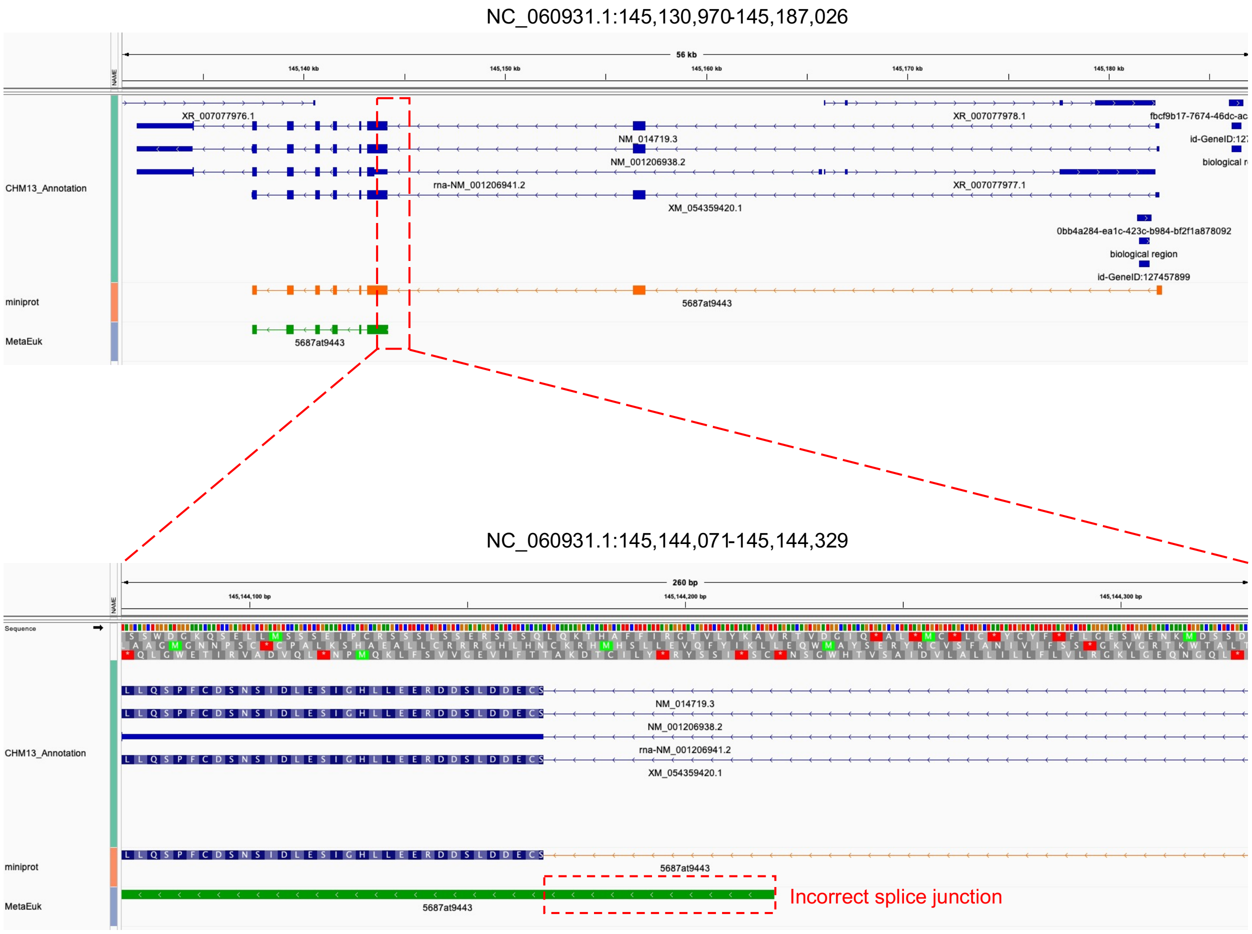

Figure S2. Example of the protein-to-genome alignment of a compleasm-specific complete gene. The upper part of the figure shows that miniprot aligns the protein sequence almost completely to the CHM13 genome, and is supported by the CHM13 annotation, while MetaEuk misses some fragments when aligning the protein sequence. The lower part of the figure shows that the reason why some fragments are missing during the MetaEuk alignment may be that MetaEuk cannot find exact splice junctions.
